# Characterizing the Rural Opioid Use Environment in Kentucky Using Google Earth: Virtual Audit

**DOI:** 10.2196/14923

**Published:** 2019-10-04

**Authors:** Natalie Danielle Crawford, Regine Haardöerfer, Hannah Cooper, Izraelle McKinnon, Carla Jones-Harrell, April Ballard, Sierra Shantel von Hellens, April Young

**Affiliations:** 1 Department of Behavioral Sciences and Health Education, Rollins School of Public Health, Emory University Atlanta, GA United States; 2 Department of Epidemiology, Emory University Atlanta, GA United States; 3 Department of Environmental Health, Rollins School of Public Health, Emory University Atlanta, GA United States; 4 Morehead State University Morehead, KY United States

**Keywords:** opioid-related disorders, rural health, built environment

## Abstract

**Background:**

The opioid epidemic has ravaged rural communities in the United States. Despite extensive literature relating the physical environment to substance use in urban areas, little is known about the role of physical environment on the opioid epidemic in rural areas.

**Objective:**

This study aimed to examine the reliability of Google Earth to collect data on the physical environment related to substance use in rural areas.

**Methods:**

Systematic virtual audits were performed in 5 rural Kentucky counties using Google Earth between 2017 and 2018 to capture land use, health care facilities, entertainment venues, and businesses. In-person audits were performed for a subset of the census blocks.

**Results:**

We captured 533 features, most of which were images taken before 2015 (71.8%, 383/533). Reliability between the virtual audits and the gold standard was high for health care facilities (>83%), entertainment venues (>95%), and businesses (>61%) but was poor for land use features (>18%). Reliability between the virtual audit and in-person audit was high for health care facilities (83%) and entertainment venues (62%) but was poor for land use (0%) and businesses (12.5%).

**Conclusions:**

Poor reliability for land use features may reflect difficulty characterizing features that require judgment or natural changes in the environment that are not reflective of the Google Earth imagery because it was captured several years before the audit was performed. Virtual Google Earth audits were an efficient way to collect rich neighborhood data that are generally not available from other sources. However, these audits should use caution when the images in the observation area are dated.

## Introduction

The opioid epidemic has had a devastating impact on Americans, resulting in increased levels of addiction and overdose, particularly among those who live in rural areas [1,2]. Although drug use has been historically perceived as an urban problem, the epidemiology of substance use and drug overdose has shifted substantially from cities to rural areas [3,4]. For example, in Kentucky alone, a state that is about 71% rural [5], synthetic opioid and heroin overdose deaths have increased to 780 deaths and 269 deaths in 2017, representing a 10-fold and 2-fold increase since 2013, respectively [6]. Moreover, the rate of drug overdose in rural areas has surpassed that of many urban areas [7,8], with Kentucky having the fourth highest overdose rate nationally [9].

Rural areas cover approximately 97% of the land area in the United States [10], and despite the salience of the physical environment for substance use and related harms in urban areas, its impact in rural areas has received significantly less attention by the scientific community [11]. There is an extensive body of literature in urban environments that has consistently shown how substance use, injection-risk behavior, and HIV transmission are related to the physical environment, which includes both built and natural elements [12-19]. This research is supported by broken windows and risk environment theories that describe how visible decay such as run-down housing in a neighborhood results in crime and disorder [20], which is consonant with the contextual environment where these risks occur [21]. In urban areas, researchers have relied on existing administrative geographical data [22], in-person audits, and, more recently, virtual audits from public data sources with video or satellite imagery to assess the physical environment [23-25].

Analysis of existing data from administrative sources such as the US Census allows researchers to bypass the efforts involved in primary data collection; however, these secondary data often fail to capture constructs that are critical for understanding many determinants of health. This is particularly true in rural and international settings where there is a unique context in which health and health behaviors are produced [26]. For example, our qualitative data suggest that self-service drive-through car washes, which are often a part of gas stations, are used as a private area for injection drug use in rural areas. Although existing business data might indicate that a gas station exists in an area, refined data on the presence of a car wash within that gas station are rarely available. In addition, administrative data sources often create measures for geographic units based on population size. Therefore, in rural areas where the population density is small, the geographic unit covers an expansive area that may lack precision and utility. Measuring characteristics of rural areas may thus require that we develop novel measures and innovative data collection strategies to fully capture the context in rural areas for the appropriate geographic exposure area.

In-person audits are commonly used when existing data sources fail to capture specific characteristics and tend to be the strongest at assessing features of the physical environment [22,27]. However, they require trained researchers who directly observe and document specific characteristics of the physical environment, and this process can be very time-consuming and costly [28,22], particularly for rural areas, which generally have a large landmass that requires long travel times [26,29]. Importantly, in-person audits in rural areas may also be limited because of poorly vascularized roads that make it difficult to identify features of the environment that are not directly off a driving path. In urban areas, there are often enough streets to connect areas that overcome this challenge.

Virtual audits using Google Earth have shown great promise in overcoming the inefficiency of in-person audits [22,26,30,31]. Google Earth is a free 3-dimensional geographic program that provides aerial, satellite, and street view imagery on the Web and covers a vast proportion of the world’s surface area [32]. Virtual *walk arounds* of an area in Google Earth have been performed systematically to capture specific characteristics of the physical environment. Recent studies using Google Earth audits have shown strong interrater reliability and concurrent validity for a number of characteristics in both national and international settings [22,33], including food environment, recreational facilities, and street characteristics. Yet, most of these studies have been conducted in urban environments, and the utility of this innovative tool for examining rural environments is unclear. In rural areas, virtual audits using Google Earth have provided measures that are comparable with in-person audits for a number of characteristics including the presence of sidewalks [34] and the number of objective housing characteristics related to healthy aging [35]. To our knowledge, no studies have examined how well Google Earth audits perform when examining characteristics that promote or reduce risks related to substance use in rural environments.

The purpose of this study was to describe the utility and reliability of performing virtual audits using Google Earth technology to measure features of the physical environment that might be related to nonmedical prescription opioid use, heroin use, injection drug use, health care use, and overdose in a rural area. We assessed interrater reliability of built environment audits using Google Earth technology and interrater reliability of Google Earth audits compared with in-person audits [36]. We use this information to make recommendations for the use of Google Earth technology in neighborhood data collection specific to rural areas.

## Methods

### Overview

Neighborhood audits via Google Earth and in-person took place within a larger study that examined the influence of the risk environment on opioid use among young adults (aged 18-35 years) in 5 counties in rural Kentucky through a partnership between the University of Kentucky and Emory University. These counties were chosen in the parent study because of high levels of nonmedical prescription opioid use, overdose, and poverty. Neighborhood audits were conducted between July 2017 and August 2018. Herein, we describe the virtual audit training, virtual audit data collection, virtual audit reconciliation, and the in-person audit.

### Virtual Audit Training

Auditors underwent extensive training before data collection. All auditors were advanced graduate students who had taken coursework in spatial data. Auditors were required to review a step-by-step protocol of the audit methods. An in-depth discussion of the definition and appropriate classification of each neighborhood characteristic was reviewed with each auditor. Independent and supervised practice audits of sample areas were then performed. Weekly discussion and troubleshooting of the data collection were performed. Additional training of the auditors was performed as needed.

### Virtual Audit Data Collection

Systematic auditing of 49 census blocks was performed for 5 rural Kentucky counties using cartographic boundary files from the 2016 US Census [37]. Census blocks are small geographical areas nested within census tracts, but larger than city blocks, for which basic demographic data can be obtained for a population. Similar to in-person neighborhood audits, Google Street View audits were systematically initiated at the same location (eg, most southeastern point) for each county to capture physical environment characteristics that might be related to nonmedical prescription opioid use, heroin use, injection drug use, health care use, and overdose among young adults living in rural areas. Previous studies have examined each block face of an area to collect the desired measures [22]. Street view images were supplemented with aerial images in Google Earth for select characteristics.

To inform the functionality of Google Earth for virtual audits in rural areas, we also collected data on the date on which the images were captured for each block to understand how well these images matched with the present time frame and in-person audits. Auditors also maintained a log of the time required to audit each block. The auditors noted when they did not know whether a location fell within a physical environment category, experienced a visual problem with Google Earth (ie, resolution issue), or if a characteristic was visible in 1 view (aerial or street view) but not in another. Each block was audited by 2 independent, trained auditors.

### Virtual Audit Reconciliation

We established a final virtual audit dataset after reconciling discrepancies between the 2 independent Google Earth auditors. Discrepancies were identified by comparing the quantity of each physical environment characteristic across the 2 independent auditors for each block. Then, a third independent Google Earth auditor, who was considered the gold standard, repeated the audit of the entire block to specifically identify the correct number of physical environment characteristics for which discrepancies were present. The third independent auditor was also able to compare the specific latitude and longitude, where the 2 independent auditors disagreed. Disagreements and reconciliation findings were reviewed daily between the third independent auditor and the senior author to finalize all discrepancies.

### In-Person Audits

To validate the data obtained from the virtual audits, in-person audits were conducted for a subsample of the census blocks where virtual audits were performed. The subsample was selected by excluding census blocks with Google Street View images that were only collected before 2015 to ensure that the neighborhood characteristics were a good representation of the current environment, given that some features of the environment can disappear and appear. Then, we further excluded the sample based on counties with at least 800 residents to ensure that there was an adequate number of physical environment characteristics for comparison with the virtual audits. A total of 8 blocks were randomly selected across all 5 counties.

We hired a resident of 1 of the counties to perform in-person audits. We provided extensive training to the in-person auditor to ensure systematic and standardized collection of each characteristic similar to the virtual audit. Driving routes were created with physical maps of each block starting at the most southeastern point of each block. A data collection tool was developed using the Fulcrum app [38], which was installed on a mobile phone to capture the latitude and longitude of each neighborhood characteristic. The Fulcrum app is ideal for rural settings where internet connectivity may be limited as it can collect data without a constant internet connection and can automatically produce time stamps of the data collection session [39].

### Physical Environment Characteristics Assessed in Virtual and In-Person Audits

Data were collected on various characteristics of the physical environment and categorized based on whether they represented land use, a health care facility, entertainment venue, or business. The specific features captured within each of these categories are shown in Textbox 1. In brief, land use characteristics are made up of features that describe the function of property, health care facilities represent buildings where an individual can obtain health care, entertainment venues represent businesses that individuals patron for pleasure, and businesses represent storefronts where individuals obtain day-to-day necessities. Importantly, categorization of each feature was based on the literature and our qualitative data that informed data collection of characteristics that were unique to the Kentucky area (eg, hollows). Existing literature has shown that land use, entertainment venues, and businesses (eg, those that allow open alcohol use or provide opportunities for sex) may promote opportunities to obtain and use illicit substances [17,40,41], whereas health care facilities may be protective and provide opportunities to reduce substance use and injection risk behaviors [13]. Given the small numbers of each feature, we combined them across each category for presentation. Disaggregated data that are deidentified for each county are available on request.

**Physical environment features assessed in virtual and in-person audits in each category.**

Land use

- Boarded up and dilapidated business [42]
- Boarded up and dilapidated homes [22,42]
- Defunct mines and industrial sites (captured using aerial views)
- Water recreation areas (captured using aerial views) [22,42]
- Hollows (captured using aerial views)
- Trailer parks (captured using aerial views)

Health care facilities

- Drug-related and HIV-related health care sites
- General health care sites [25]
- Pharmacies [22]
- Syringe/needle exchange programs

Entertainment venues

- Liquor store [22,43]
- Tobacco store
- Motel/hotel

Businesses

- Fast food restaurants [22,44]
- General restaurants [44]
- Gas stations [22,44]
- Car washes
- Cemeteries (captured using aerial views)
- Truck stops

### Data Analysis

First, we calculated the frequencies of the images collected each year. Audit time from the virtual audits was summed for each block and averaged across all blocks in each county. We describe the median and interquartile range (IQR) for each physical environment category. Owing to the sparsity of the features measured in these rural communities and the small number of census blocks, traditional intraclass correlations (ICCs) were unstable and vastly underestimated between-auditor agreement [45,46]. Therefore, we calculated the percent agreement of each audit based on complete agreement between the 2 independent auditors. 

We also calculated the percent at which each auditor overestimated or underestimated each feature based on the virtual gold standard. If an auditor identified the same number of a feature within a given block as the gold standard, this auditor’s scoring was coded a *match*. If the auditor identified a higher or lower number of a feature than the gold standard, the auditor’s scoring was coded *over* and *under*, respectively. The percentages were calculated as number of segments for which an auditor identified *over*, *under*, or *matched* with the gold standard. Percent agreement was also reconciled between the final virtual audit data, compared with the gold standard auditor, and the in-person audits.

## Results

Textbox 1 describes each feature that was collected for each physical environment category in the virtual and in-person audits. A total of 533 data points were captured in the virtual audits, 383 (72%) of which were from images taken before 2015 with more than 40% of those dating back to 2009 (data not shown). Table 1 shows the median and IQR for each characteristic captured in the final (reconciled) virtual audit data. In general, the data for these rural areas are sparse. There was a median of 14 (IQR 9-25) features in each block in the land use category. Boarded up and dilapidated homes (median 8, IQR 3-8) were the most frequently observed feature in the land use category and overall. There was a median of 0 (IQR 0-1) health care facilities per block, where each feature was equally underrepresented in each block. There was a median of 1 (IQR 1-3) entertainment venue where each feature within this category was also equally sparse. There was a median of 6 businesses (IQR 2-9), which was mostly represented by cemeteries (median 2; IQR 1-2). There were no syringe/needle exchange programs identified in any county, and liquor stores were only present in 1 county, which was the only county where alcohol could be sold in our geographic sample.

The median number of Google Earth images that were difficult to decipher because of poor resolution of the images or missing street view images where aerial images were available was also small, but the disaggregated data showed that missingness was more common in the least populated areas. The average audit time per block was 1.97 hours, ranging from 58 min to 227 min per block. In-person audits required about 8 hours of auditing time per block, not including the time required to map each area.

Table 2 shows the reliability of the physical environment characteristics for the virtual and in-person audits. The reconciled data comparing the 2 independent virtual audits with the gold standard show that agreement was high for health care facilities (>83%), entertainment venues (>95%), and businesses (>61%). Agreement was poor for land use features (>18%) and varied substantially between auditors (36.7% vs 18.4%). Land use features included boarded up and dilapidated homes, businesses and defunct mines and industrial sites, and hollows. The independent auditors tended to underestimate (range: 2%-73.5%) versus overestimate (range: 0%-8.2%) the presence of a feature compared with the gold standard. For features in the land use category, these overestimates were high (range: 61.2%-73.5%), whereas health care facilities (range: 10.6%-16.7%), entertainment venues (range: 2%-4.1%), and businesses (range: 18.4%-34.7%) had lower estimates.

When comparing the reconciled virtual audit data with the in-person audit data, reliability was high for health care facilities (83%) and entertainment venues (62%) but was poor for land use (0%) and businesses (12.5%). The in-person audit data generally underestimated features in the land use category (75%) and health care facilities (16.7%) compared with the reconciled virtual data. However, features in the entertainment venue and business categories were overestimated (25% and 62.5%, respectively) compared with the reconciled virtual data.

**Table 1:** Descriptive neighborhood characteristics collected using virtual audits for 5 counties in Kentucky (N=64,061).

Characteristics	All counties (n=64)
Land use, mean (IQR^a^)	14 (9-25)
Health care facilities, mean (IQR)	0 (0-1)
Entertainment venues, mean (IQR)	1 (1-3)
Businesses, mean (IQR)	6 (2-9)
Do not know, mean (IQR)	1 (0-4)
Unclear, mean (IQR)	0 (0-0)
Missing images, mean (IQR)	2 (0-3)
Virtual audit time per block (hours), mean (SD)	1.97 (1.07)
In-person audit time per block (hours), mean (SD)	8 (1.92)

^a^IQR: interquartile range.

**Table 2:** Reliability of neighborhood characteristics collected in rural Kentucky using virtual and in-person audits versus the gold standard.

Characteristics		Virtual audits		In-person audits, % agreement
		Audit 1, % agreement	Audit 2, % agreement	
**Land use**				
	Over	2.0	8.2	25.0
	Match	36.7	18.4	0.0
	Under	61.2	73.5	75.0
**Health care facilities**				
	Over	0.0	0.0	0.0
	Match	89.4	83.3	83.0
	Under	10.6	16.7	16.7
**Entertainment venue**				
	Over	2.0	0.0	25.0
	Match	95.9	95.9	62.5
	Under	2.0	4.1	12.5
**Businesses**				
	Over	6.1	4.1	62.5
	Match	75.5	61.2	12.5
	Under	18.4	34.7	25.0

## Discussion

### Principal Findings

Virtual audits using Google Earth show strong potential for assessing the built environment in rural areas for objective features such as health care facilities, entertainment venues, and businesses. However, virtual audits are less reliable when the characteristic requires some judgment or may change within a short period such as dilapidated housing or features that are no longer in operation. Other studies have similarly encountered difficulties when assessing characteristics of the built environment that are more ambiguous, such as the amount of loitering on the street or graffiti [26,47]. In our study, virtual audit reliability was the lowest for the land use category, which included boarded up and dilapidated homes and businesses. This may reflect the difficulty in capturing subjective features that require some judgment to assess. However, the aerial imagery in Google did allow for better view of mines, which would have been difficult, if not impossible, to assess in street view. Thus, the flexibility of street and aerial imagery is a strength.

### Challenges and Limitations

Our audits in rural environments revealed some unique challenges that should be considered in future virtual audits. First, older and missing images in Google Earth are an important limitation. Google states that street view audits are performed every 2 to 3 years to maintain updated imaging [33]. However, a substantial proportion (72%) of the images we analyzed were taken before 2015 and dated as far back as 2008, 9-10 years before the virtual audit was done. In these instances, specific issues were that the street view data were unavailable or incomplete, and for some characteristics, such as strip malls, they were also unavailable in the aerial view. It is unclear if Google Earth fails to update their images in places with smaller populations or other characteristics, but we found older images in all of the counties assessed in our virtual audit regardless of how populated the county was. Older images made it difficult to estimate reliability between our virtual and in-person audits. For example, lower agreement may reflect images that were not captured by an auditor because of auditor failure, or it could reflect that a characteristic appeared or disappeared in the years since the Google Earth image was taken. To attempt to reduce this bias, we performed in-person audits in census blocks with images captured post 2015. However, it is still possible that homes or businesses can become run-down or be torn down completely within just a couple of years [48]. In line with this hypothesis, the in-person auditor tended to report an underestimate of features in the land use category and overestimate of features in the business category. Of note, our audits could only identify features that were visible through external signage of the feature. In many areas, health services such as syringe exchange are often not advertised widely to avoid negative attention. Therefore, syringe exchange programs that were located in health departments were not captured through our audits. It is important to note that this would also be a limitation of in-person audits.

By their very nature, rural environments will produce fewer observations than urban environments for many features that research shows are relevant to substance misuse and related harms. However, we must note that as research on physical environment and drug use expands, new influential features of rural areas may be identified that are more commonplace in these areas. The sparsity of the features assessed here poses difficulty when calculating standard reliability measures. ICCs severely underestimate agreement when the data are sparse [45,46]. Therefore, we were only able to quantify percent agreement, but these estimates may also be affected by the small sample size.

In addition, Google Earth frequently crashed, which resulted in loss of work. Furthermore, auditor fatigue may be particularly salient in rural audits because rural areas have fewer features to assess, so the audits may be more cumbersome. Rural areas also generally have a larger landmass than urban areas and require more time to thoroughly review than an urban area. This may contribute to auditor turn over, resulting in the need for additional training and resources for new auditors.

Nevertheless, virtual audits are an efficient way to collect rich neighborhood data that are not routinely available through other sources. Google Earth is particularly viable in rural areas where there is a need to capture features that are distant from roads that are less connected than urban areas and would be difficult if not impossible to reach via in-person audits. Thus, Google Earth is a powerful tool that should be considered for future research and data collection in rural environments. These data will allow for research that elucidates key features of rural environments related to opioid use and points to critical points of intervention to reduce substance misuse and related harms.

## Abbreviations

ICC: intraclass correlation
IQR: interquartile range
