# Correction: Characterizing the Rural Opioid Use Environment in Kentucky Using Google Earth: Virtual Audit

**DOI:** 10.2196/21442

**Published:** 2020-08-12

**Authors:** Natalie Danielle Crawford, Regine Haardörfer, Hannah Cooper, Izraelle McKinnon, Carla Jones-Harrell, April Ballard, Sierra Shantel von Hellens, April Young

**Affiliations:** 1 Department of Behavioral Sciences and Health Education Rollins School of Public Health Emory University Atlanta, GA United States; 2 Department of Epidemiology, Emory University Atlanta, GA United States; 3 Department of Environmental Health Rollins School of Public Health Emory University Atlanta, GA United States; 4 Morehead State University Morehead, KY United States

The authors of “Characterizing the Rural Opioid Use Environment in Kentucky Using Google Earth: Virtual Audit” (J Med Internet Res 2019;21(10):e14923), the authors noticed four errors in the contributing authors' names and degrees.

Firstly, the name of the second author was misspelled and has been revised from

Regine Haardöerfer

to

Regine Haardörfer.

Next, the degree listed for Hannah Cooper has been revised from:

Hannah Cooper, PHD

to

Hannah Cooper, SCD

Then, the degree listed for April Ballard has been revised from:

April Ballard, BA

to

April Ballard, MPH

Lastly, the academic degrees listed for Carla Jones-Harrell were incorrect. Her degree listing has been revised from:

Carla Jones-Harrell, MPhil

to

Carla Jones-Harrell, MUEP, MPH.

These corrections will appear in the online version of the paper on the JMIR website together with the publication of this correction notice. Because this change was made after submission to PubMed, PubMed Central, and other full-text repositories, the corrected article has also been resubmitted to those repositories.

